# LINE-1 Hypomethylation in Cancer Is Highly Variable and Inversely Correlated with Microsatellite Instability

**DOI:** 10.1371/journal.pone.0000399

**Published:** 2007-05-02

**Authors:** Marcos R.H. Estécio, Vazganush Gharibyan, Lanlan Shen, Ashraf E.K. Ibrahim, Ketan Doshi, Rong He, Jaroslav Jelinek, Allen S. Yang, Pearlly S. Yan, Tim H-M. Huang, Eloiza H. Tajara, Jean-Pierre J. Issa

**Affiliations:** 1 Department of Leukemia, University of Texas M. D. Anderson Cancer Center, Houston, Texas, United States of America; 2 Department of Pathology, University of Cambridge, Cambridge, United Kingdom; 3 Department of Medicine, University of Minnesota, Minneapolis, Minnesota, United States of America; 4 Department of Medicine, University of Southern California, Los Angeles, California, United States of America; 5 Human Cancer Genetics Program, Ohio State University Comprehensive Cancer Center, Columbus, Ohio, United States of America; 6 Department of Molecular Biology, Faculdade de Medicina de São José do Rio Preto (FAMERP), São José do Rio Preto, São Paulo, Brazil; Max Delbrueck Center for Molecular Medicine, Germany

## Abstract

**Background:**

Alterations in DNA methylation in cancer include global hypomethylation and gene-specific hypermethylation. It is not clear whether these two epigenetic errors are mechanistically linked or occur independently. This study was performed to determine the relationship between DNA hypomethylation, hypermethylation and microsatellite instability in cancer.

**Methodology/Principal Findings:**

We examined 61 cancer cell lines and 60 colorectal carcinomas and their adjacent tissues using LINE-1 bisulfite-PCR as a surrogate for global demethylation. Colorectal carcinomas with sporadic microsatellite instability (MSI), most of which are due to a CpG island methylation phenotype (CIMP) and associated MLH1 promoter methylation, showed in average no difference in LINE-1 methylation between normal adjacent and cancer tissues. Interestingly, some tumor samples in this group showed increase in LINE-1 methylation. In contrast, MSI-showed a significant decrease in LINE-1 methylation between normal adjacent and cancer tissues (P<0.001). Microarray analysis of repetitive element methylation confirmed this observation and showed a high degree of variability in hypomethylation between samples. Additionally, unsupervised hierarchical clustering identified a group of highly hypomethylated tumors, composed mostly of tumors without microsatellite instability. We extended LINE-1 analysis to cancer cell lines from different tissues and found that 50/61 were hypomethylated compared to peripheral blood lymphocytes and normal colon mucosa. Interestingly, these cancer cell lines also exhibited a large variation in demethylation, which was tissue-specific and thus unlikely to be resultant from a stochastic process.

**Conclusion/Significance:**

Global hypomethylation is partially reversed in cancers with microsatellite instability and also shows high variability in cancer, which may reflect alternative progression pathways in cancer.

## Introduction

Cancer is a complex disease, which arises from both genetic and epigenetic errors. The importance of genetic alterations in cancer, including chromosome abnormalities and genetic mutations as well its causative factors (e.g. ionizing radiation and chemical carcinogens) are now well known. The epigenetic component of cellular transformation, however, was until recently poorly understood. It has been known for decades that genome-wide hypomethylation happens in tumors compared to normal cells [1]–[4] and overexpression of oncogenes was postulated to be a result of this hypomethylation. DNA hypermethylation in cancer gained attention a few years later with studies from Baylin et al. [5], [6] and Jones et al. [7]. The latter alteration occurs in CpG island promoters of single-copy genes and impairs gene transcription, resulting in silencing of tumor supressor genes. Several studies described a tissue-specific pattern of methylation in cancer and hundred of targets genes are known, including tumor suppressor genes and genes involved in invasion, angiogenesis and apoptosis [8], [9]. The age-related nature of promoter hypermethylation in normal tissues [10] has been proposed as a predisposition factor in cancer.

An important and unsolved question is whether genome-wide hypomethylation and single-copy CpG island promoter hypermethylation are two independent alterations or if they are mechanistically linked. Unbiased studies of DNA methylation changes have identified both frequent hypermethylation and hypomethylation in several types of neoplasia [11]–[14]. Attempts to answer this question resulted in contradictory findings, with some groups supporting [15], [16] and others refuting [17], [18] a link between both alterations.

Here, we conducted a genome-wide methylation study in cancer cell lines and primary tumors to determine the relationship between DNA hypomethylation, hypermethylation and microsatellite instability in cancer. The retrotransposable element LINE-1 was used as a surrogate of genome-wide hypomethylation, and methylation microarrays expanded our analysis to other classes of repetitive elements. Genome-wide methylation differed in colorectal carcinomas belonging to distinct CpG island methylation phenotype (CIMP) groups, most notably in the ones with associated microsatellite instability (MSI), where hypomethylation was infrequent compared to both CIMP+/MSI-and CIMP-/MSI-groups. Cancer cell lines exhibited a large variation in genome-wide demethylation, which was tissue-specific and thus unlikely to be a stochastic process. In summary, our results show that genome-wide hypomethylation in cancer is highly variable, the causes of which are unknown, and the existence of a strong inverse link between global hypomethylation and microsatellite instability in cancer.

## Materials and Methods

### Tissue samples and cell lines

Sixty matched pairs of tumor and apparently normal adjacent colon specimens were obtained from patients treated at Johns Hopkins University (Baltimore, MA). CpG island methylation phenotype (CIMP) and microsatellite analysis were previously determined for these samples [19]. Peripheral blood lymphocytes were obtained from five healthy donors, and normal colon mucosa tissue was ressected from five individuals submitted to surgery for gun shot wounds or non-malignant lesions. This study was approved by the Ethics Committee of Johns Hopkins University (Baltimore, MA), and informed consent was obtained from all participants.

Sixty-one cancer cell lines from eight different tissues (breast, central nervous system, colon, leukemia, liver, lung, ovary and prostate) were obtained from the American Type Culture Collection (ATCC, Manassas, VA) and cultured using standard methods. DNA from patients and cell lines was extracted using standard phenol–chloroform extraction methods.

### Bisulfite-pyrosequencing LINE-1 analysis

Bisulfite treatment was performed as reported [20]. Methylation analysis of LINE-1 promoter (GenBank accession number X58075) was investigated using a pyrosequencing-based methylation analysis. We carried out 50 µl PCR in 60 mM Tris–HCl pH 8.5, 15 mM ammonium sulfate, 2 mM MgCl2, 10% DMSO, 1 mM dNTP mix, 1 unit of Taq polymerase, 5 pmol of the forward primer (5′-TTTTTTGAGTTAGGTGTGGG-3′), 5 pmol of the reverse-biotinylated primer (5′-BIO-TCTCACTAAAAAATACCAAACAA-3′) and 50 ng of bisulfite-treated genomic DNA. PCR cycling conditions were 95°C for 30 s, 50°C for 30 s and 72°C for 30s for 50 cycles. The biotinylated PCR product was purified and made single-stranded to act as a template in a pyrosequencing reaction as recommended by the manufacturer using the Pyrosequencing Vacuum Prep Tool (Pyrosequencing, Inc., Westborough, MA). In brief, the PCR product was bound to Streptavidin Sepharose HP (Amersham Biosciences, Uppsala, Sweden) and the Sepharose beads containing the immobilized PCR product were purified, washed, denatured using a 0.2 M NaOH solution, and washed again. Then, 0.3 µM pyrosequencing primer (5′-GGGTGGGAGTGAT-3′) was annealed to the purified single-stranded PCR product and pyrosequencing was performed using the PSQ HS 96 Pyrosequencing System (Pyrosequencing, Inc.).

### Methylated CpG island amplification (MCA)/CpG island microarray

Sixteen colorectal tumors were compared to their normal appearing adjacent tissue using a CpG island microarray protocol developed in our laboratory. For each sample, MCA amplicons were produced according Toyota et al. [20] using RXMA PCR adaptors. To minimize amplification bias due to differential incorporation of fluorescent dyes, we opted for an indirect-labeling protocol. For this, the incorporation of amino-allyl dUTP (aa-dUTP, Sigma) into 600 ng each of tumor DNA and normal DNA was conducted using the Bioprime DNA-labeling system protocol (Life Technologies). Cy5 and Cy3 fluorescent dyes were coupled to aa-dUTP-labeled tumor and normal adjacent amplicons, respectively, and cohybridized to the HCGI12K-Human CpG 12K Array (Microarray Centre, University Health Network, Toronto, Canada). Hybridization and post-hybridization washing procedures are according to DeRisi and colleagues and can be found at http://www.microarrays.org. Hybridized slides were scanned with the GenePix 4000A scanner (Axon Instruments, Foster City, CA) and the acquired images were analyzed with the software GenePix Pro 6.0. Only spots with annotated DNA sequence with 90% or more of their length overlapping a repetitive element were used for analysis. A total of 770 spots representing repetitive DNA of different classes were evaluated using this method and the methylation data for each spot was represented as the log_2_ratio of tumor (Cy5, red)/normal (Cy3, normal) intensities. Values≥1.0 (2-fold change) were indicative of increased methylation (hypermethylation) and values≤−1.0 were indicative of decreased methylation (hypomethylation) in tumor. Unsupervised hierarchical clustering was done using the program CIMminer (http://discover.nci.nih.gov/cimminer/) with calculation for distance using absolute correlation and complete linkage clustering.

### Statistical analysis

The significance of the differences observed between means was estimated using two-sided Student's t-test. P value of less than 0.05 was considered statistically significant. One-way ANOVA was used when comparing similarity for three or more groups. Statistical analyses were carried out with Statistica software package (StatSoft, Tulsa, OK).

## Results

### LINE-1 methylation in colorectal carcinomas correlates with MSI status

**Figure 1:**
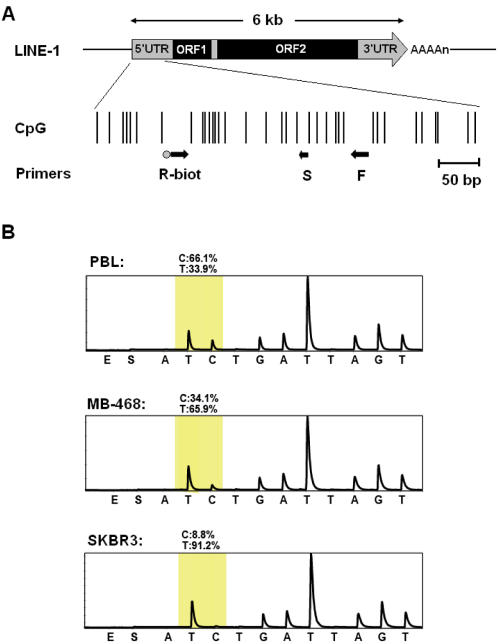
Quantitation of DNA methylation using bisulfite LINE-1 PCR and pyrosequencing. A) Diagram of the CpG island promoter (GenBank accession no. X58075, nucleotide position 108–520 bp) associated with the full length LINE-1. Each vertical line represents a single CpG site. The 3′UTR, 5′UTR and two ORFs of LINE-1 are shown at the top. Arrows indicate the location of primers used for bisulfite PCR (R-biot and F) and pyrosequencing (S). B) Representative LINE-1 pyrograms for normal peripheral blood lymphocytes (PBL) and breast cancer cell lines (MB-468 and SKBR3). The pyrogram quantitates C for methylated and T for unmethylated DNA. The shaded region represents the CpG site quantitated in LINE-1 elements, and the percent methylation is shown above the peak.

We applied the pyrosequencing method to determine the methylation density in the LINE-1 promoter. In previous studies, we validated the application of this method to evaluate genome-wide methylation content [21], [22] and showed a strong positive correlation between LINE-1 methylation and LC-MS (liquid chromatography/mass spectrometry) data. Thus, LINE-1 methylation levels can be used as a surrogate of genome-wide demethylation. The map of the LINE-1 promoter with primers and probe positions is presented in Figure 1A. This method relies on bisulfite treatment of DNA which modifies unmethylated cytosines to tymidines while methylated cytosines are non-reactive. PCR of bisulfite-treated DNA results in pools of products containing both methylated and unmethylated DNA that can be discriminated and quantitated using the pyrosequencing method. Representative LINE-1 pyrograms are presented in Figure 1B. We investigated five normal colon mucosa and five peripheral blood lymphocyte (PBL) DNA samples from healthy donors to determine the normal levels of LINE-1 methylation. The LINE-1 methylation was similar in these two different tissues, with an average of 71.9% in PBL and 70.8% in normal colon mucosa.

We next evaluated LINE-1 methylation in sixty primary colorectal carcinomas and their normal matching mucosa and correlated this with demographic, clinopathologic and molecular variables (Table 1). Colorectal tumors averaged 54.9% methylation (SEM = 1.1%) versus 64.3% (SEM = 0.5%) methylation in adjacent normal tissue, corresponding to an average relative demethylation of 14.6% (P<0.001). Compared to LINE-1 methylation in normal colon, which averaged 70.8% (SEM = 1.3%), both tumor and adjacent to tumor colon mucosa were demethylated, with a respective average of 22.5% and 9.2% relative demethylation (P = 0.001). No differences in LINE-1 methylation were found by age or gender, but a significant difference was found for side, with lower levels of methylation in normal adjacent right colon (63.0%) compared to left colon (65.5%, P = 0.016) and stage, with lower levels of methylation for tumors in stages 3 and 4 (51.9%) compared to stages 0 to 2 (57.1%, P = 0.028).

**Table 1:** Methylation density of LINE-1and clinical and demographic characteristics of 60 colorectal carcinomas and their normal appearing adjacent mucosa*

Variable	N	Normal			Cancer		
		Mean	95% CI	P^a^	Mean	95% CI	P
**Subjects**							
*Patients*		64.3	63.3–65.3	0.001	54.9	52.7–57.2	0.001
*Controls*		71.5	68.9–74.0				
**Age**							
*60 and younger*	13	62.8	60.3–65.3	0.142	53.8	49.0–58.7	0.595
*more than 60*	47	64.7	63.7–65.7		55.2	52.6–57.9	
**Gender**							
*male*	44	64.3	63.3–65.3	0.989	55.1	52.3–57.9	0.738
*female*	16	64.3	61.9–66.7		54.4	50.6–58.2	
**Side** b							
*left*	24	65.5	64.2–66.8	0.016	56.8	53.5–60.0	0.173
*right*	26	63.0	61.4–64.6		53.2	50.6–56.8	
**Stage**							
*0 to 2*	35	65.0	63.7–66.2	0.139	57.1	54.5–59.7	0.028
*3 and 4*	21	63.4	61.6–65.2		51.9	48.0–55.8	
**MSI status**							
*negative*	50	64.6	63.6–65.7	0.117	53.8	51.3–56.3	0.004
*positive*	10	62.6	60.1–65.1		60.6	56.8–64.4	
**CIMP status**							
*negative*	27	63.9	62.5–65.3	0.458	52.5	48.9–56.2	0.057
*positive*	33	64.6	63.3–66.0		56.9	54.2–59.6	
**CIMP/MSI**							
*CIMP+/MSI+*	10	62.6	60.1–65.1	0.087	60.6	56.8–64.4	0.038
*CIMP+/MSI-*	23	65.5	63.9–67.1		55.3	51.7–58.8	
*CIMP-/MSI-*	27	63.9	62.5–65.3		52.5	48.9–56.2	

* Means of cancer methylation is significantly (P<0.001) lower than mean of adjacent normal for all categories except for MSI+cancers (p = 0.24)

a Significant P values (<0.05) are underlined

b Side information was not available for all cases

CI = confidence interval

**Figure 2:**
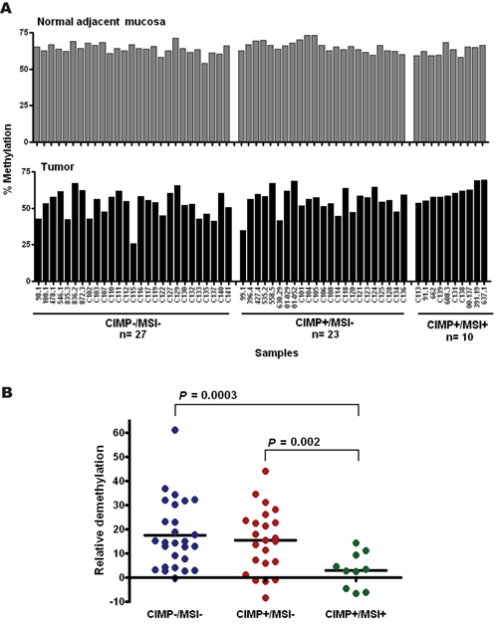
Differential LINE-1 methylation among CIMP/MSI groups in primary colorectal carcinoma samples (CRCs). A) Colorectal tumor DNA and their normal appearing adjacent mucosa from sixty patients were evaluated for LINE-1 methylation. These tumors were previously evaluated for CpG island methylator phenotype (CIMP), using a panel of single-copy genes methylation analysis, and microsatellite instability (MSI) status, resulting in the identification of three CIMP/MSI groups. In normal appearing mucosa (top) little variation in LINE-1 methylation is observed between samples and CIMP/MSI groups (average methylation = 64.3%), while in tumor (bottom) several samples undergo high LINE-1 demethylation (25/60 tumor samples have methylation density bellow 55%), most notable in CIMP+/MSI-and CIMP-/MSI-groups. B) Relative LINE-1 demethylation in CRCs. Relative demethylation was calculated as the percent change of LINE-1 methylation in tumor compared to its normal appearing mucosa. Both CIMP+/MSI-and CIMP-/MSI-samples presented in average 16% demethylation for LINE-1, while no significant changes were observed for the CIMP+/MSI+samples. For the CIMP+group, 4–9% increase of methylation density for LINE-1 was observed for a small fraction of samples, most of them identified as CIMP+/MSI+samples.

The primary colorectal tumors presented a high variation in LINE-1 methylation among different samples (Figure 2A), and the stratification of these colorectal tumors and their normal adjacent tissue reveals a non-uniform variability in LINE-1 methylation. CRC with sporadic microsatellite instability (MSI), most of which are due to MLH1 promoter methylation, showed no difference in LINE-1 methylation between normal adjacent and cancer tissues (62.6%±1.1% versus 60.6%±1.7%, P = 0.33), with an average decrease in methylation of only 3.12%±2.3%. By contrast MSI-cases had a significant decrease in LINE-1 hypomethylation between normal adjacent and cancer tissues (64.6%±0.5% versus 53.8%±1.2%, P<0.0001). Apparently, LINE-1 hypomethylation was independent from CIMP status, since CIMP+/MSI-cases and CIMP-cases were equally hypomethylated (15.4%±2.7% versus 17.7%±2.7%, P = 0.56). This unequal distribution of relative demethylation by presence of microsatellite instability is represented in the Figure 2B, which illustrates the maintenance of LINE-1 methylation in CIMP+/MSI+tumors compared to normal appearing mucosa, while CIMP+/MSI-and CIMP-/MSI-undergo severe hypomethylation, with one case presenting an extreme relative demethylation (61.3%).

### Methylation analysis of repetitive elements using MCA/CpG island microarrays

**Figure 3:**
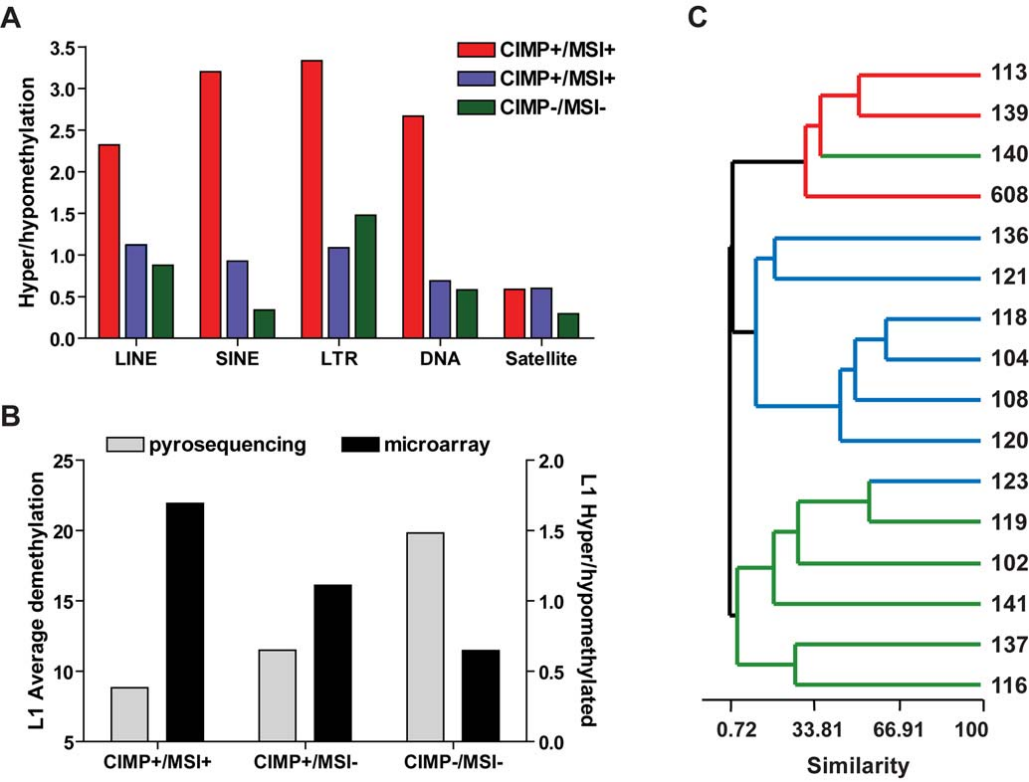
Methylated CpG Island Amplication (MCA)/CpG island microarray for repetitive DNA sequences. A) Relative abundance of hypermethylated and hypomethylated repeats for each CIMP/MSI group. A higher number of hypermethylated compared to hypomethylated repeats was observed for the CIMP+/MSI+group, and a gradual change in representation of hypermethylated and hypomethylated repeats was seen for the CIMP+/MSI-and CIMP-/MSI-groups, resulting in an overrepresentation of hypomethylated repeats in microsatellite stable groups. B) Validation of microarray results for LINE repeats. Note that CIMP/MSI groups with higher demethylation, as determined by bisulfite-pyrosequencing of LINE-1, presented also a higher number of hypomethylated LINE repeats by microarray analysis, as represented by a lower hyper/hypomethylation ratio. C) Unsupervised hierarchical clustering was applied to methylation data from a set of 770 repetitive DNA sequences across sixteen colorectal tumors paired with their normal appearing mucosa DNA. The colorectal tumors dendrogram is shown, and the sample ID for each case is included in the right. The terminal branches are color coded to represent the CIMP/MSI status of the tumor sample (red, CIMP+/MSI+; blue, CIMP+/MSI-; green, CIMP-/MSI-). Overall, samples of the same CIMP/MSI group clustered together, reinforcing the different methylation fate for repetitive DNA sequences methylation in each group. LINE, long interspersed nuclear elements; SINE, short interspersed nuclear elements, LTR, long terminal repeats; DNA repeats; Satellite repeats.

In addition to LINE-1 methylation analysis by bisulfite PCR and pyrosequencing, we also evaluated the methylation status of repetitive elements including LINE (long interspersed nuclear elements), SINE (short interspersed nuclear elements), LTR (long terminal repeats), DNA and satellite repeats, by coupling MCA (methylated CpG island amplification; 20) to a CpG island microarray containing a total of 770 spots representing repetitive DNA of different classes. The first analysis was performed by counting hypermethylated (log_2_ratio<1.0) and hypomethylated (log_2_ratio<−1.0) repeats separated according to their different classes (Figure 3A). For the CIMP+/MSI+samples, each one of the repeats classes except satellite repeats were found to be enriched for hypermethylation in tumor DNA compared to normal adjacent mucosa (hypermethylation/hypomethylation = 2.4-fold in average). The enrichment for hypermethylated sequences decreased sharply in CIMP+/MSI-and CIMP-/MSI-(0.88-and 0.71-fold, respectively). These findings suggest that there is a strong pressure for maintenance and/or de-novo methylation of repetitive elements in the MSI+group. Validation of our microarray method was done by comparing the results for LINE-1 to pyrosequencing data in the same colorectal samples. This analysis revealed that tumors with the lowest LINE-1 demethylation by pyrosequencing analysis showed the highest enrichment for hypermethylated LINE repeats (Figure 3B), with the inverse situation being observed for tumors with the highest LINE-1 demethylation. These results support that our microarray analysis is a suitable technique to access methylation changes in repetitive elements.

Finally, using the normalized log_2_ratio values of individual spots, we performed unsupervised hierarchical clustering to reveal the similarities among the 16 colorectal tumor cases studied. The resulting clustered image map showed a good concordance with the expected segregation of individual samples by known CIMP/MSI status (Figure 3C), suggesting that the methylation signatures of these tumors are not restricted to single-copy genes but also involve repetitive DNA elements.

### Hypomethylation of LINE-1 in cancer cell lines shows tissue-specific variability

**Figure 4:**
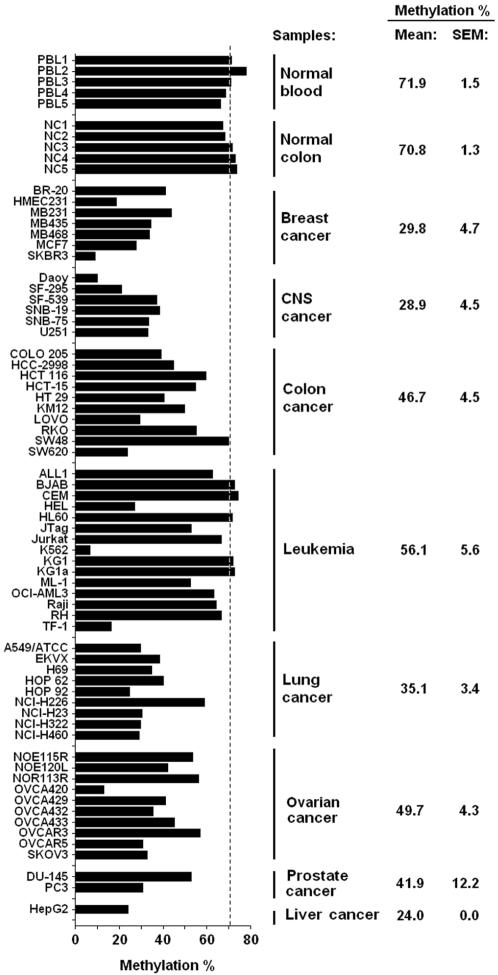
LINE-1 methylation variability in cancer cell lines. DNA samples of normal peripheral blood lymphocyte, normal colon mucosa and sixty-one cell lines from eight different tissues types were investigated for LINE-1 methylation using bisulfite PCR followed by pyrosequencing. The normal tissues presented high levels of LINE-1 methylation (above 70% in average), and a large variation in methylation levels was observed for cancer cell lines, with a minimum methylation density of 6.5% being observed for the leukemia cell line K562. Taken as a group, leukemia cell lines were moderately demethylated (average 56.1%), followed by ovary, colon, prostate and lung cancer cell lines (variation from 49.7% to 35.1%). Central nervous system (CNS), breast and the one liver cancer cell lines tested were deeply demethylated (bellow 30% in average). Dotted line represents average methylation in normal controls.

To verify if the variability in genome-wide methylation is restricted to primary colorectal carcinomas or also occurs in other tumor types, we applied the LINE-1 bisulfite-pyrosequencing method to sixty-one cancer cell lines from eight different tissues types (breast, central nervous system, colon, leukemia, lung, ovary, prostate and liver). Interestingly, we found a marked decrease in LINE-1 methylation in most of the studied cell lines (Figure 4). Overall, 50/61 tested cancer cell lines were hypomethylated for LINE-1, with a relative demethylation of 15% or more (absolute methylation density lower than 60%) compared to peripheral blood lymphocytes and normal colon mucosa. Similarly to primary colorectal tumors, these cancer cell lines exhibited high variability in LINE-1 methylation, ranging from 6.5% (K562, a CML cell line with erythroleukemia features) to 74.2% (CEM, an acute lymphoblastic leukemia cell line). There is an apparent tissue-specificity for demethylation; the lowest levels of LINE-1 methylation were observed in liver (24.0%), followed by CNS (28.9%), breast (29.8%), lung (35.1%), prostate (41.9%), ovary (49.7%), colon (46.7%) and leukemia (56.1%). While an interesting finding, we caution generalization of the data because: (i) LINE-1 methylation was not studied for normal tissues except colon and peripheral blood; and (ii) a small number of cell lines were analyzed for liver and prostate cancer.

Another significant finding is that some cell lines show extreme hypomethylation. While leukemias in general present LINE-1 methylation levels equal to PBL, 3/15 cell lines have more than 50% relative demethylation (K562, HEL and TF-1). A similar situation is observed in other tissues, were “demethylation champions” cell lines were identified (SKBR3 in breast and OVCA420 in ovarian). An attractive explanation is that genes involved in DNA methylation maintenance are missing or mutated in these cell lines.

## Discussion

DNA methylation plays an important role in normal cells, being involved in X-chromosome inactivation, imprinting and repression of repetitive elements such as retrotransposons and endogenous retroviruses [23], [24]. At the same time, CpG islands in the promoter region of single-copy genes are methylation-free, which is important to allow transcription. In cancer, a reverse scenario is found, with single-copy CpG island hypermethylation and genome-wide hypomethylation. The aim of the present work was to determine the relationship between these two abnormal events, using cancer cell lines from several tissue-types and primary colorectal tumors as a model.

While genome-wide demethylation and single-copy CpG island hypermethylation occur in cancer, it is poorly understood whether these two alterations are linked. Our data based on methylation levels of repetitive elements, using both a specific assay for LINE-1 methylation analysis and a microarray platform comprising almost 800 repetitive DNA sequences from different classes, show that those tumors with the highest levels of aberrant hypermethylation (CIMP+/MSI+), also showed the lowest levels of genome-wide hypomethylation, compared to normal adjacent mucosa. Indeed, these tumors showed frequent increase in methylation of repetitive elements, as revealed by both LINE-1 and microarray analysis. Interestingly, CIMP+/MSI-and CIMP-/MSI-showed higher levels of LINE-1 hypomethylation, reinforcing the uniqueness of CIMP+/MSI+tumors. Although, CIMP+/MSI-and CIMP-/MSI-groups were equally hypomethylated for LINE-1, suggesting that microsatellite instability is the main molecular alteration associated with lack of LINE-1 hypomethylation. These findings are concordant with previous reports of lack of global hypomethylation in microsatellite unstable tumors [15]. The consensus interpretation of these data is that colorectal tumors arise from two distinct progression pathways: global hypermethylation with microsatellite instability and global hypomethylation with chromosome instability. However, it is necessary to note that the CIMP+/MSI+group is not only characterized by microsatellite instability, but also for a higher frequency of hypermethylated CpG islands. Indeed, the causative factor of the observed microsatellite instability is the exclusive hypermethylation of MHL1 in these tumors, and other genes like p16 and THBS1 are also found more frequently methylated in CIMP+/MSI+compared to CIMP+/MSI-[25]. In addition, the microarray analysis of repetitive DNA supported the existence of a group of tumors (CIMP+/MSI+) under strong pressure to de novo methylation of both CpG island promoters and repetitive elements and re-classified colorectal tumors into their known CIMP/MSI groups. Notably, CIMP+/MSI-and CIMP-/MSI-were mostly clustered apart, suggesting that the microarray analysis revealed some special features of each group not seen by LINE-1 bisulfite-pyrosequecing. For example, SINE repeats show a gradual change in hypermethylation/hypomethylation according the CIMP/MSI, with CIMP+/MSI-being an intermediate group. Also, satellite repeats (mainly represented by centromeric and pericentromeric repeats) showed a more stable pattern of methylation and it maybe related to their functional role in chromosome segregation during cell division. By contrast, Ehrlich et al. [18] found that hypomethylation and hypermethylation are independent in ovarian cancers, based in the capacity of these alterations to predict the degree of malignancy in ovarian tumors. However, direct comparisons of hypomethylation in cancers with and without high levels of methylation (i.e. CIMP) were not studied. More studies are necessary to answer why difference repetitive elements classes have different susceptibility to DNA hypomethylation. In general, our microarray data suggest that some classes of repetitive elements can be subject to the same methylation pressures exerted on CpG islands on CIMP+cancers.

Using LINE-1 methylation as a surrogate for global demethylation, we found a large variation in methylation levels between different cancer cell lines, with tissue specificity. Some tissues like breast, CNS and lung undergo marked LINE-1 demethylation in cancers in a fairly homogeneous fashion. In other tested tissues, like colon and leukemia, some cell lines had methylation levels similar to those exhibited by normal colon and blood tissues, while others were profoundly demethylated. Although a follow-up study including normal samples from the same studied tissues is required to confirm this observation, an analysis performed by Chalichagorn et al. [26] did not showed a marked difference in methylation between normal samples from various tissues. Similarly to our results, a previous study by Florl et al. [27] had found a marked difference between bladder and renal carcinomas, with only the first exhibiting LINE-1 demethylation. The large variation in global hypomethylation observed implicates non-stochastic mechanisms for this defect, and also suggests a selective advantage for tumors with severe hypomethylation. Indeed, recent experiments show that tumor formation is induced in mice after global genomic hypomethylation [28], [29]. Using conditional transgene technology to reduce expression of DNMT1, Gaudet et al. [28] observed spontaneous formation of T-cell lymphomas with acquisition of additional genomic changes. Holm et al. [29] generated mice that mimic loss of imprinting (LOI), presumed to be due to hypomethylation, and in these animals tumor formation was also observed. The causes of such differential demethylation among cancer of different tissues are unknown, and both genetic and exposure factors may play roles in this. Profound hypomethylation, as observed in the cell line K562 and others could be related to specific loss of function of genes that control methylation of repetitive elements. Candidate genes are those coding for proteins that have been described to exert function as “heterochromatin guardians”. For example, the LSH protein, a member of the SNF2/helicase family proteins, is required for genome-wide methylation. Knockout mice for the Lsh gene displayed perinatal mortality and showed marked demethylation of repetitive elements that is independent from alterations in RNA levels of DNMT1 [30].

In summary, our results show that genome-wide hypomethylation is highly variable in cancer cells, as is single-copy CpG island hypermethylation. Both alterations can be found in the tumors and each one can promote tumorigenesis by independent processes. Our study also provides evidence for a strong inverse link between global hypomethylation and microsatellite instability in cancer.
